# Transcriptome analysis of the impact of diabetes as a comorbidity on tuberculosis

**DOI:** 10.1097/MD.0000000000031652

**Published:** 2022-12-30

**Authors:** Tao Liu, Yaguo Wang, Jing Gui, Yu Fu, Chunli Ye, Xiangya Hong, Ling Chen, Yuhua Li, Xilin Zhang, Wenxu Hong

**Affiliations:** a Shenzhen Center for Chronic Disease Control and Prevention, Shenzhen, China; b Key Laboratory of RNA Biology and National Laboratory of Biomacromolecules, CAS Center for Excellence in Biomacromolecules, Institute of Biophysics, Chinese Academy of Sciences, Beijing, China; c Guangdong TB Healthcare Co., Ltd., Foshan, China; d The Fourth People's Hospital of Foshan City-Foshan Tuberculosis Prevention and Control Institute, Foshan, China.

**Keywords:** comorbidity, diabetes, PBMC, transcriptome, tuberculosis

## Abstract

Diabetes mellitus patients with pulmonary tuberculosis (DMTB) comorbidity has been recognized as a major obstacle towards achieving the World Health Organization goal of reducing the tuberculosis incidence rate by 90% in 2035. Host immune responses affected by diabetes can lead to increased susceptibility, severity and poor treatment outcomes in DMTB patients, and the underlying mechanisms have not yet been fully elucidated. This study aimed to identify key immunological and cellular components that contribute to increased morbidity and mortality in DMTB cases.

**Methods:**

We performed RNA-Seq of total RNA isolated from peripheral blood mononuclear cells from 3 TB, 3 diabetes mellitus, and 3 DMTB patients and healthy controls, and analyzed differential expression, pathway enrichment and clustering of differentially-expressed genes (DEGs) to identify biological pathways altered specifically in DMTB patients.

**Results:**

Bioinformatic analysis of DEGs suggested that enhanced inflammatory responses, small GTPases, the protein kinase C signaling pathway, hemostasis and the cell cycle pathway are likely implicated in the pathogenesis of the DMTB comorbidity.

**Conclusion:**

The DMTB comorbidity is associated with an altered transcriptome and changes in various biological pathways. Our study provides new insights on the pathological mechanism that may aid the development of host-directed therapies for this increasingly prevalent disease in high TB burden countries.

## 1. Introduction

As the world’s most deadly infectious disease, tuberculosis (TB) claims more than a million lives each year and affected 8.9–11.0 million people in 2019 according to the World Health Organization.^[1]^ Although the global TB incidence rate and the number of TB deaths have been falling in recent years, the soaring prevalence of diabetes mellitus (DM) worldwide and the fact that DM greatly increases TB susceptibility and severity has meant that the diabetes mellitus patients with pulmonary tuberculosis (DMTB) comorbidity has become a major global public health concern and has posed a great challenge to TB control and eradication.^[1–4]^ Countries with the highest TB burden tend to be those with the largest number of diabetes cases.^[4–6]^ In 2019 an estimated 0.35 million TB patients (9–17% of all TB cases) had diabetes. Diagnosis of TB in diabetic individuals is more challenging,^[7]^ and mounting evidence shows that TB infection in diabetes patients leads to more unfavorable outcomes than in non-diabetic individuals. A recent meta-analysis of 44 studies showed that type 2 diabetes, the most prevalent form of DM, increases the risk of developing TB by two- to four-fold.^[8–10]^ Moreover, patients with DMTB comorbidity are more likely to show more low-field pulmonary lesions, more cavitary lesions and more extrapulmonary lesions.^[11,12]^ Additionally, as reported by several observational studies, DM can cause adverse TB treatment outcomes, including delays in pathogen clearance, treatment failure, relapse and recurrence.^[13–18]^ The rapidly rising prevalence of diabetes, the increased risk and severity of DMTB comorbidity, and poor treatment outcomes emphasize the critical need to target this population in TB control programs.

Current view of the most likely cause of the increased susceptibility and severity of TB in DM patients is that hyperglycemic condition leads to compromised host immune responses and function.^[2,19]^ However, the underlying mechanisms have not yet been fully understood. Most evidence suggests that the abnormal accumulation of glucose intermediates and excessive reactive oxidative species are the major factors impacting on the function of the host immune system.^[20,21]^ In order to investigate how DM adversely affects TB patients and identify specific immunological pathways or biological processes underpinning DMTB comorbidity, in the present study we analyzed enriched pathways in differentially-expressed genes (DEGs) that displayed increased expression from DM to DMTB and in DEGs that are uniquely present in the DMTB patients relative to HCs. Cell cycle pathway, small GTPases and molecules in the PKC signaling pathway were identified here for the first time to be potentially involved in the pathogenesis of DMTB comorbidity. Our findings may contribute to the elucidation of novel disease biomarkers, pathogenic mechanisms and therapeutic targets.

## 2. Materials and Methods

### 2.1. Patient recruitment

Adults with newly diagnosed, bacteriologically confirmed pulmonary TB with and without diabetes were recruited between April 2021 and June 2021 at Shenzhen Center for Chronic Disease Control and Prevention (Shenzhen, China). Patients were classified as having DM if HbA1c was ≥6.5% or plasma glucose levels were ≥200 mg/dL 2 hour post-challenge in the 75 g oral glucose tolerance test. The active TB group included individuals who displayed TB-specific clinical symptoms, had abnormal chest radiography consistent with active TB, and acid-fast bacilli sputum smear positive and/or bacterial culture positive. All patients were either newly diagnosed or under anti-TB or anti-diabetic treatment for no longer than 2 weeks at the time of recruitment. Individuals taking immunosuppressants or with other comorbidities were excluded. Informed consent was obtained from all subjects. This study was approved by the Ethical Committee of Shenzhen Center for Chronic Disease Control and Prevention (NO: SZCCC-2021-030-01-YJ).

### 2.2. Isolation of peripheral blood mononuclear cells (PBMCs) and RNA extraction

Peripheral blood (10 mL) from each participant was withdrawn from the median ubital vein of the antecubital fossa in Sodium citrate vacutainer tubes (SANLI, China). PBMCs were separated by density gradient using Lympholyte Cell Separation Media (TBD, China) within 4 hours of blood withdrawal. The number of viable cells was counted using Automated Cell Counter (Count star, China) by trypan blue staining. The PBMCs from each subject were adjusted to a density of 1–4 × 10^6^ cells/mL in TRIzol reagent (Thermo Fisher) and stored at –80°C until they were used for total RNA extraction.

Total RNA was extracted from each sample using TRIzol reagent (Invitrogen Life Technologies®, Grand Island, NY). The isolated RNAs were digested with Dnase I (Invitrogen™, Waltham, MA) to remove the residual DNA, and were suspended in 25 μL of DNase/RNase-free water. Total RNA from each sample was quantified and its quality was assessed by Agilent 2100 Bioanalyzer (Agilent Technologies, Palo Alto, CA), NanoDrop (Thermo Fisher Scientific Inc., Waltham, MA), and 1% agarose gel electrophoresis. Total RNA (1 μg) with an RNA integrity number above 8 was used for library preparation.

### 2.3. Library preparation and paired-end sequencing

RNA libraries for next generation sequencing were constructed with nine patients and three HCs according to the manufacturer’s protocol (NEBNext® Ultra™ Directional RNA Library Prep Kit for Illumina®, San Diego, CA). The rRNA was removed from the total RNA samples using Ribo-Zero™ rRNA removal Kit (Human/Mouse/Rat) (Illumina) and the remaining RNA was then fragmented and reverse-transcribed. The first strand cDNA was synthesized using ProtoScript II Reverse Transcriptase with random primers and actinomycin D. The second-strand cDNA was synthesized using Second Strand Synthesis Enzyme Mix (including dACG-TP/dUTP). The double-stranded cDNA was purified by AxyPrep Mag PCR Clean-up (Axygen) and then treated with End Prep Enzyme Mix to repair both the ends. dA-tailing was performed in one reaction, which was followed by T-A ligation to add adaptors to both the ends. Size selection of adaptor-ligated DNA was then performed using AxyPrep Mag PCR Clean-up (Axygen), and ~360 bp fragments (with an approximate insert size of 300 bp) were recovered. Libraries with different indices were multiplexed and loaded on an Illumina HiSeq instrument according to the manufacturer’s instructions (Illumina). The libraries were sequenced on an Illumina HiSeq X Ten platform and 150 bp paired-end reads were generated. About 7G raw reads for each sample were generated. The transcriptome reads have been deposited in the NCBI SRA database with accession numbers PRJNA767116.

### 2.4. RNA-seq data analysis

To obtain high-quality reads, the reads containing adaptor sequences, more than 10% ambiguous bases (noted as N) and low-quality bases (Qphred ≤20 bases account for more than 50% of the entire read length of the reads) were filtered. The resulting clean reads were then aligned to the human reference genome (GRCh38 genome) using HISAT2 with the default values.^[22]^ Transcript assembly was performed using Cufflinks v2.1.1 with the default parameters.^[23]^ All transcripts were compared with gene models in the reference genome to identify novel genes expressed from previously intergenic regions (class code “u”) using Cuffcompare.^[24]^ The gene expression levels in all the samples were calculated using fragments per kilobase of exon model per million mapped reads, and the genes with more than 1 fragments per kilobase of exon model per million mapped reads in at least one sample were used for further analysis.^[25]^ An adjusted *P* value (FDR < 0.05) and fold change (FC) ratio (|Log2 FC| ≥ 1) were used to determine the DEGs between patients and health control using DESeq R package.^[26]^ Principal component analysis (PCA) was performed using the DESeq package for clustering the samples based on gene expression patterns.^[26]^ The heatmap of DEGs was clustered using pheatmap^[27]^ (version 1.0.8, http://cran.r-project.org/web/packages/pheatmap) package in software (version 3.6.1).^[28]^

### 2.5. Functional enrichment and bioinformatics analysis

The Metascape^[29]^ (http://metascape.org) online database integrates over forty bioinformatics knowledge bases enabling the extraction of abundant annotations, as well as the identification of enriched pathways and the construction of protein–protein interaction networks from lists of gene and protein identifiers. Using the Gene Ontology (GO) and the Kyoto Encyclopedia of Genes and Genomes (KEGG) tools within Metascape, we analyzed a genes list containing DEGs genes to further explore the biological process of disease. The GO analysis included three categories: biological process (BP), cellular component, and molecular function. We undertook enrichment analysis of DEGs different comparison groups. Protein–protein interaction (PPI) analysis was carried out to predict the interaction of selected genes based on the STRING Database (V.11).^[30]^ Further, Molecular Complex Detection (MCODE) algorithm was used to check modules of the PPI network.^[31]^ KEGG pathway and GO terms with computed P values ˂ 0.05, were considered significantly enriched based on the hypergeometric distribution.

### 2.6. Cluster analysis

Cluster analysis of gene expression data from the different patient was performed using Mfuzz v.2.46.0.^[32]^ The software package implements a method for clustering short time-series expression data that can differentiate between real and random patterns of temporal gene expression changes and assigns each gene to the model profile that most closely matches the temporal gene expression profile for that gene as determined by the correlation coefficient. Mfuzz v.2.46.0 was used to detect different subclustering models of gene expression among the different groups and also incorporates GO enrichment functionality for biological interpretation of time-series gene expression data.

### 2.7. Statistical analysis

The differentially expressed mRNAs between HC and the other three groups (DM, TB, DMTB) were analyzed using the *t*-test. Significance was defined as *P* < .05. All statistical analysis was performed using R software (version 3.6.1).^[28]^

### 2.8. Data availability

All raw and processed RNA files are available from the NCBI’s Sociological Research in America database (ID accession number PRJNA767116, https://www.ncbi.nlm.nih.gov/sra/PRJNA767116).

## 3. Results

### 3.1. Overall study design

**Figure 1.**
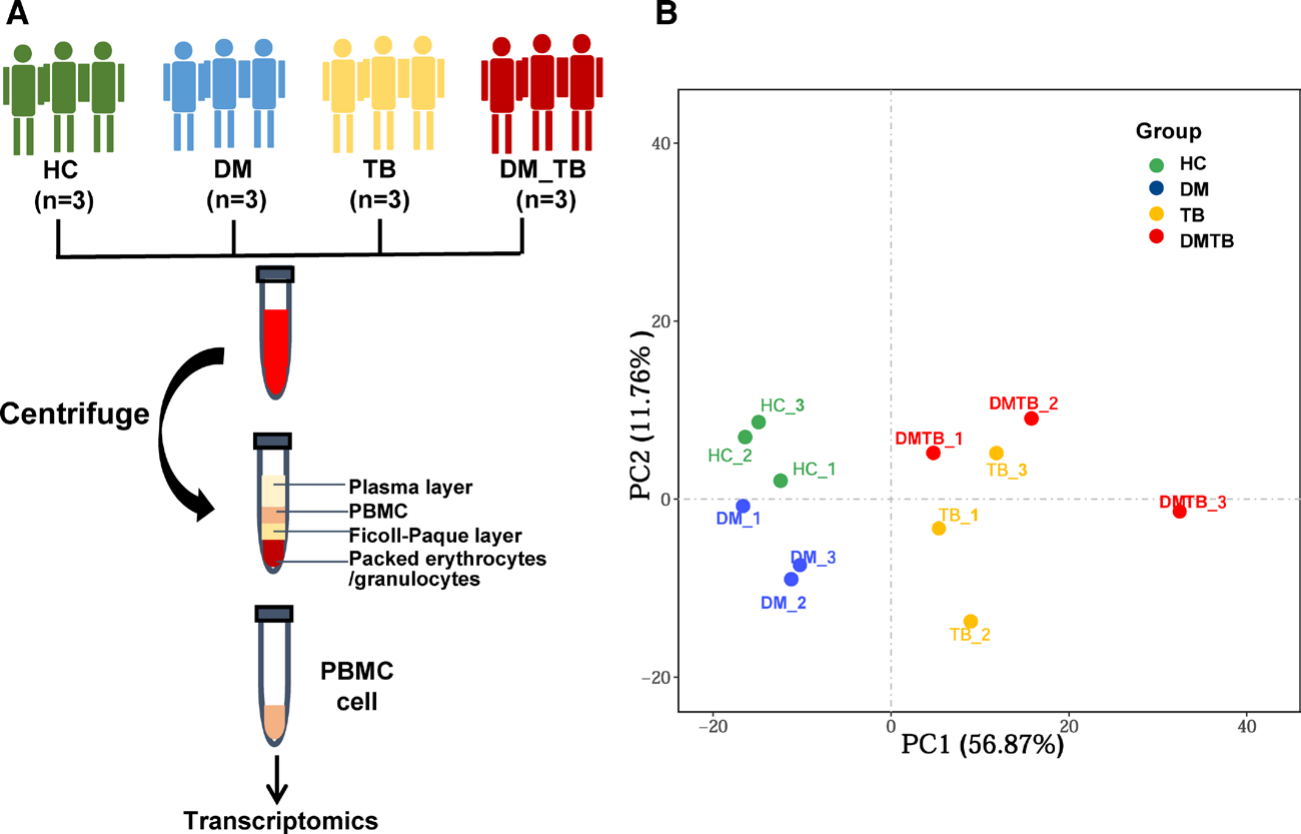
Overview of study design. (A) Schematic summary of the study design and patient cohort. (B) PCA plot of the transcriptomics data (13,269 quantified genes) of the study participants. PCA = principal component analysis.

In this study, we performed RNA-Seq of total RNA isolated from PBMCs to identify biological pathways altered specifically in DMTB patients. PBMC are mononuclear cells in Peripheral blood, including lymphocytes and mononuclear cells. PBMC cells were low amounts in whole blood samples and alterations in gene expression during disease development and progression in cells present at low amounts in whole blood could thus not be easily detected. Here, we isolated PBMCs from the study subjects using Ficoll-hypaque density gradient centrifugation and analyzed transcriptome profiles specific to those cells (Fig. 1A). We recruited 3 DMTB comorbid patients, 3 TB patients, 3 DM patients and 3 healthy individuals from Shenzhen Center for Chronic Disease Control and Prevention (Shenzhen, China). There was no statistically significant difference in the ages of subjects in these subgroups.

### 3.2. RNA-seq summary statistics

Deconvolution and filtering of sequence reads to remove adaptor-dimer contamination yielded a mean of 48.69 ± 1.25 million reads per individual barcoded RNA-seq sample library (n = 12 libraries). These filtered reads were then aligned to the human genome (GRCh38). This yielded a mean of 47.69 ± 1.2 million filtered reads (94.11%) that uniquely mapped to this human genome; a mean of 1.66 ± 0.19 million reads (3.47%) that mapped to multiple genomic locations. Filtering of the RNA-seq data using 12 samples (including TB patients, DM patients, DMTB patients and healthy control) produced 17,242 genes suitable for downstream differential expression analysis among them, 13,269 genes were retained for analysis after filtering (see Table S1, Supplemental Content, which show the RNA-seq summary statistics and he details of the genes). Data exploration was carried out using principal components analysis and hierarchical cluster analysis. PCA of quantified genes (13,269 genes) revealed that there were significant altered in Disease group (including DM, TB and DMTB groups) compared with those of the healthy controls (Fig. 1B). In particular, the patients of DMTB comorbidity were more closely related to patients with TB and the HC and DM individuals tended to cluster together, so it was speculated that patients of DMTB comorbidity were greatly affected by TB (Fig. 1B).

### 3.3. Differential expression gene analysis

**Figure 2.**
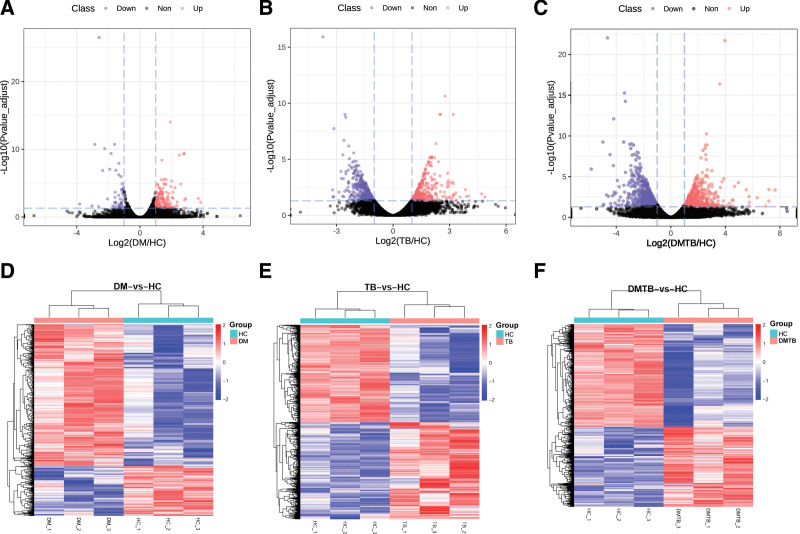
Transcriptomic changes in DM, TB and DMTB patients relative to healthy controls. (A–C) Volcano plot of different expressed genes (DEGs) for DM, TB and TB-DM patients relative to healthy controls. Gene expression profiles of DM (n = 3, B), TB (n = 3, C) and DMTB (n = 3, D), each relative to healthy controls (n = 3). Dashed lines: cutoff values (|fold change| >2 and an adjusted *P* value < 0.05). Red dots:The gene of up-regulation, Purpledots: the gene of down-regulation. Red dot or Purpledots in the volcano plot correspond to the genes whose expression was significantly changed, and gray dots show genes without significant expression change or with expression fold change < 2. (D–F) Heatmap visualization of significantly different expressed gene for DM, TB and DMTB patients relative to healthy controls. The graphs show the relative intensity of differentially expressed gene. Genes included in the heatmap meet the requirement that fold change >2 or <0.5 and adjusted *p*-value (*t* test) of < 0.05 and. Color bar represents the relative intensity of detected gene from −2 to 2. DM = diabetes mellitus, DMTB = diabetes mellitus patients with pulmonary tuberculosis, TB = tuberculosis.

Differential expression was tested by the pair-wise comparison: DM versus HC, TB versus HC and DMTB versus HC. The gene with significant changes in expression level in the pair-wise comparison (fold change ≥2 or fold change ≤ 0.5 and *P* values < .05) were presented in Figure 2 and Table 1. A total of 182 differentially expressed genes were identified between the DM and HC group, of which 136 were up-regulated and 46 were down-regulated (Fig. 2A). Meanwhile, there were 845 differentially expressed genes between the TB and HC group, with 471 of which were down-regulated and 374 were up-regulated (Fig. 2B). The difference between DMTB patients and healthy people was the largest. And there are 1516 differentially expressed genes, of which 582 were up-regulated and 934 were down-regulated (Fig. 2C), which indicated that the biological processes in DMTB group and healthy people were significantly different. Unsupervised cluster analysis and PCA indicated that the differentially expressed gene generated by the pair-wise comparisons could reflect the different statuses of diseases (Fig. 2D–F, and see Fig.S1, Supplemental Content, which demonstrates PCA plot of DEGs for DM, TB and DMTB patients relative to healthy controls).

**Table 1:** The result of different expressed genes (DEGs).

Compare	Upregulated	Down-regulated	Total DEGs
DM vs HC	136	46	182
TB vs HC	374	471	845
DMTB vs HC	582	934	1516

DEGs = differentially-expressed genes, DM = diabetes mellitus, DMTB = diabetes mellitus patients with pulmonary tuberculosis, HC = healthy controls, TB = tuberculosis.

### 3.4. GO and KEGG analysis of DEGs

**Figure 3.**
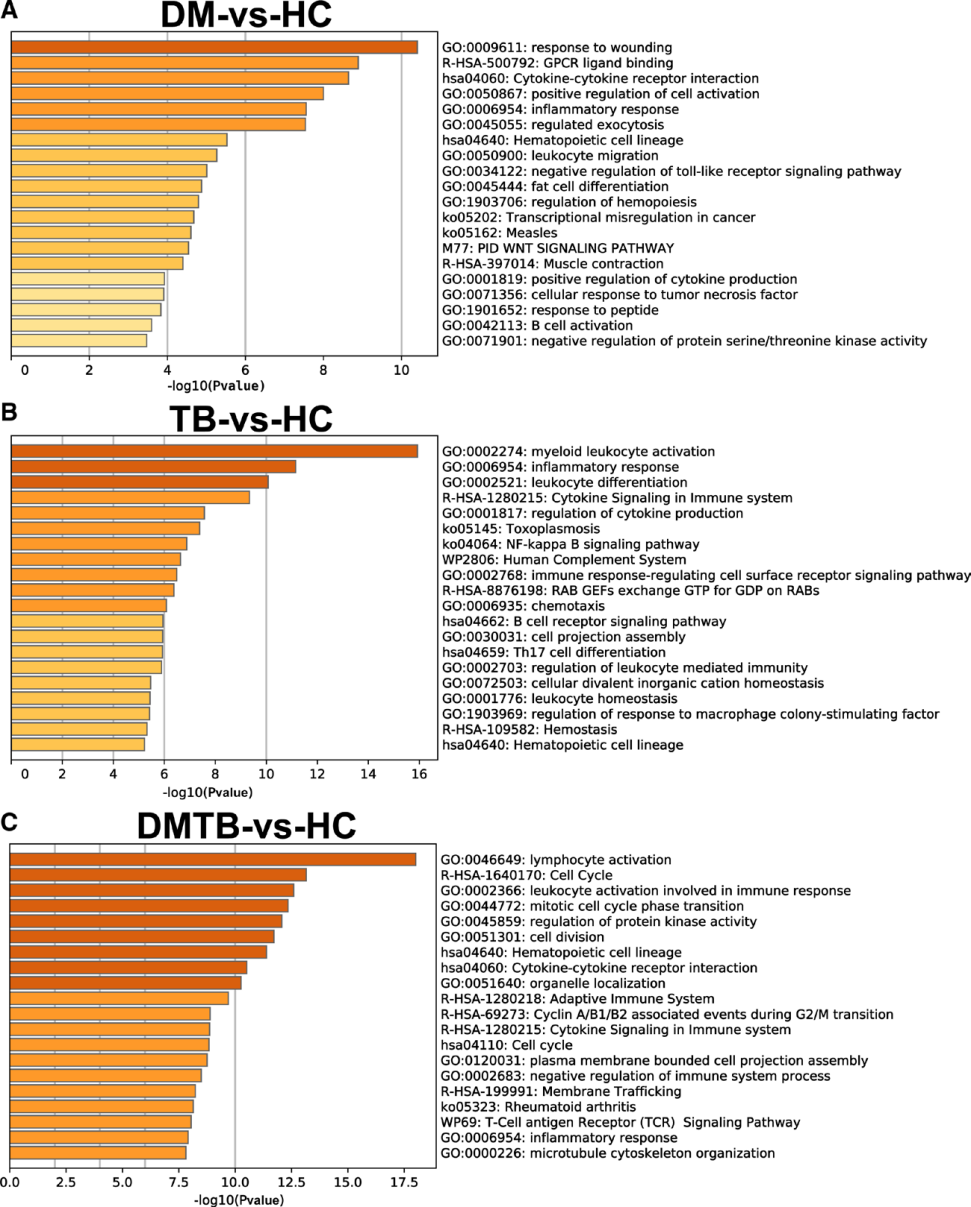
Heatmap of enriched terms across DEGs from different comparison groups in metascape. (A–C) Heatmap of enrichment terms across DEGs for DM, TB and TBDM patients relative to healthy controls, color by *P* value. Top 20 most significantly enriched function terms in DM versus HCs (A), TB versus HCs (B) and DM_TB versus HCs (C). DEGs = differentially-expressed genes, DM = diabetes mellitus, DMTB = diabetes mellitus patients with pulmonary tuberculosis, TB = tuberculosis.

To further understand the mechanism and function of DEGs, gene ontology (GO), BP and Reactome gene sets were applied using Metascape (Fig. 3 and see Table S2, Supplemental Content, which show the enrichment analysis of GO biological processes and KEGG for DEGs in DM and healthy). Then, we performed GO and KEGG analysis on the 182 DEGs in comparison between DM and HC. As shown in Figure 3A. Among the top 20 clusters with their representative enriched terms, there were 13 items of GO biological process, five items of KEGG pathway and two items of Reactome Gene Sets, based on DEGs from compared with DM and healthy group. The results showed that the DEGs from compared with DM and healthy group were mainly involved in BP such as response to wounding, inflammatory response and regulated exocytosis (Fig. 3A).

And in comparison between TB and HC, we performed GO and KEGG analysis on the 845 DEGs. The enrichment results of DEGs that these genes are mainly involved in cellular immunity functions such as myeloid leukocyte activation, leukocyte differentiation and Cytokine Signaling in Immune system. The KEGG pathways that these genes were involved in were NF-kappa B signaling pathway, B cell receptor signaling pathway, and Th17 cell differentiation (Fig. 3B, and see Table S3, Supplemental Content, which show the enrichment analysis of GO biological processes and KEGG for DEGs in TB and healthy).

Finally among the top 20 clusters with their representative enriched terms, there were 10 items of GO biological process, four items of KEGG pathway and six items of Reactome Gene Sets, based on DEGs from compared with DMTB co-infection and healthy group. The main functions of the differential genes are similar to those of the TB group, mainly related to cellular immunity such as lymphocyte activation, leukocyte activation involved in immune response and cell division (Fig. 3C, and see Table S4, Supplemental Content, which show the enrichment analysis of GO biological processes and KEGG for DEGs in DMTB and healthy). These results are similar to the previously reported results,^[33]^ indicating that our results are highly credible.

### 3.5. Clustering analysis of the DEGs

**Figure 4.**
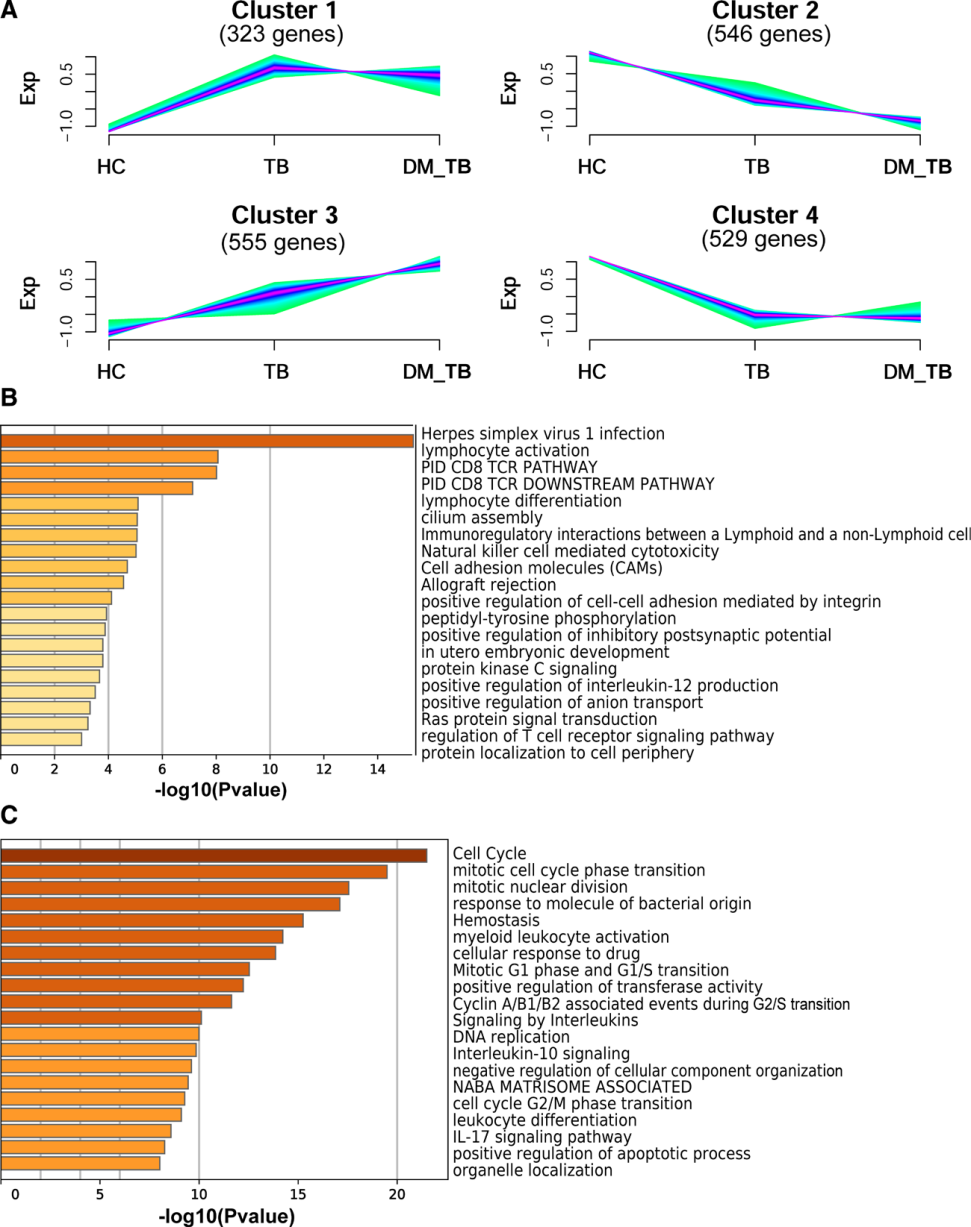
Expression profiles were analyzed according to gene expressions between HC, DM and DMTB groups. (A) Expression patterns of DEGs were determined based on FPKM values using the Mfuzz package and grouped into four different clusters. Colors varying from green to purple represent that the trends of genes become more suitable to the changes of the cluster. (B and C) Heatmap of enriched terms across the down-regution genes (Cluster 1, B, including 546 different expressed genes) and upregulation genes (Cluster 3, C including 555 different expressed genes). two-sided hypergeometric test, *P* value < .05.

The Mfuzz package is used to carry out cluster analysis, and 4 typical clusters are achieved (Fig. 4A) using the total of 1963 DEGs derived from all three comparisons. In cluster 1 (323 genes), expressions of the genes were upward from HC to TB, and were almost stable from TB to DMTB. In cluster 2 (546 genes), an increasing expression trend was observed from HC to DMTB. In cluster 3 (555 genes), gene expressions declined from HC to DMTB and then leveled out from TB to DMTB. In cluster 4 (529 genes), a continual decrease in gene expression was presented from HC to TB, and were almost stable from TB to DMTB (Fig. 4A). Gene expression levels in clusters 2 and 3 showed significant change from TB to DMTB, indicating that genes in those two clusters may be those that lead to the exacerbated conditions in DMTB patients when DM comes into play. With further studying clusters 2 and 3, we found that the genes that continued to decline were mainly involved in regulation of T cell receptor signaling pathway, lymphocyte activation and lymphocyte differentiation (Fig. 4B). The enrichment analysis of cluster3 genes showed that the continuously up-regulated genes are mainly involved in the biological processes related to cell cycle regulation such as cell cycle, mitotic cell cycle phase transition and response to molecule of bacterial origin (Fig. 4C, and see Fig. S2A, Supplemental Content, which show the network of enriched terms). Moreover, to further investigate the relationship between Co-infective and important molecular, a PPI network was constructed according to information from the Metascape online database. PPI network and MCODE components identified in the gene lists are shown in Figure S2 (see Fig. S2, Supplemental Content, which show enrichment analysis of the continuously up-regulated genes in HC-TB-DMTB (Cluster3)) and the top three modules with high scores were obtained from the PPI network. The biological functions mainly related to MCODE 1 were negative regulation of cellular component organization, and response to iron ion or metal ion, and in MCODE 2 these were resolution of sister chromatid cohesion, mitotic anaphase, and mitotic metaphase, anaphase. While in MCODE 3 these were Clathrin-mediated endocytosis, Membrane Trafficking, and Vesicle-mediated transport (see Fig. S2B, Supplemental Content, which PPI network and MCODE components identified in the gene lists).

### 3.6. Study on differential expressed genes in patients with DMTB

**Figure 5.**
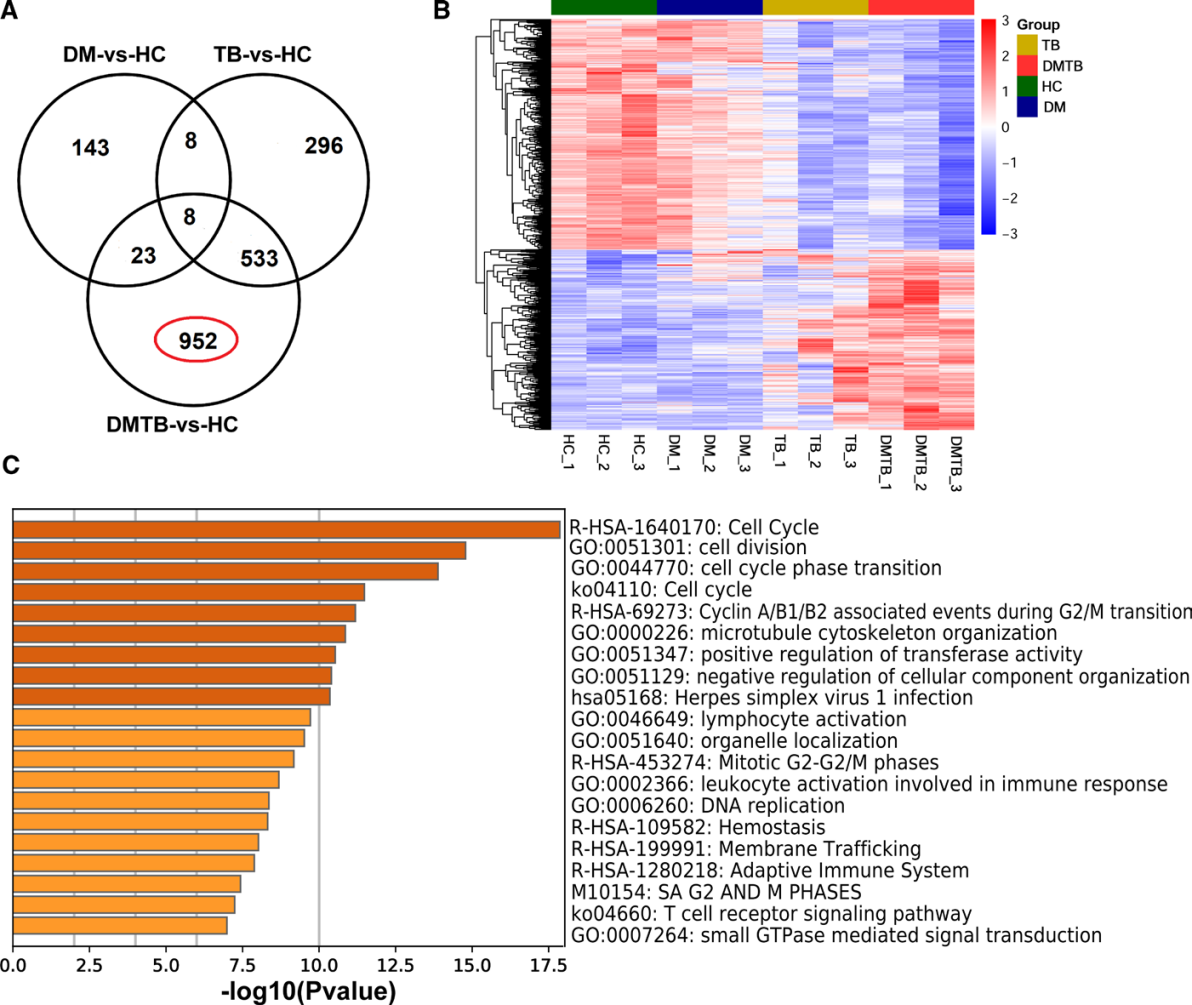
Analysis of the enriched pathways of the 952 DEGs uniquely present in the DMTB versus HC comparison. (A) Venn diagram of all the DEGs identified in the study. The number of DEGs in common to two or more comparisons or unique to only one comparison is shown. (B) Heatmap visualization of the 952 DEGs uniquely present in the DMTB versus HC comparison. (C) Histogram showing the Gene Ontology and KEGG classifications of the 952 DEGs uniquely present in the DMTB versus HC comparison. DEGs = differentially-expressed genes, DM = diabetes mellitus, DMTB = diabetes mellitus patients with pulmonary tuberculosis, TB = tuberculosis.

Compared with healthy people, DMTB patients have more differentially expressed genes (1516 DEGs), of which 952 are only found in DMTB patients (Fig. 5A). From the cluster analysis of 952 differential genes, it can be seen that the expression levels of these genes in the TB group and DM group are also different from those of healthy people (Fig. 5B). To gain insight into the underlying mechanism of the DMTB comorbidity, we examined the biological pathways specifically enriched in these 952 DEGs. The most significantly enriched pathways, similar to those enriched in cluster 3, were related to “cell cycle,” “small GTPase mediated signal transduction,” “hemostasis” and immunological pathways (Fig. 5C).

## 4. Discussion

Due to the rising prevalence of DM, especially in countries with highest TB burdens, DMTB comorbidity has become a major challenge of TB elimination. However, the mechanisms by which diabetes undermines host immune responses and TB clearance have not yet been clearly elucidated. To investigate the effects of diabetes on the development of DMTB comorbidity, we performed RNA sequencing of PBMCs from TB, DM and DMTB patients and HCs, and used bioinformatics to identify biological pathways underlying the increased susceptibility and severity of DMTB patients. Our results suggested that aside from enhanced inflammatory responses and the hemostasis pathway reported in previous studies,^[33,34]^ small GTPases, PKC signaling pathway, and the cell cycle pathway were also likely implicated in the pathogenesis of the DMTB comorbidity.

Most prior transcriptome studies of TB and the DMTB comorbidity have used whole blood samples, and alterations in gene expression during disease development and progression in cells present at low amounts in whole blood, such as monocytes, T-cells and B-cells, could thus not be easily detected. Here, we isolated PBMCs from the study subjects and analyzed transcriptome profiles specific to those cells.

Pathways most enriched in TB patients compared to HCs included: “hematopoietic cell lineage,” “cytokine-cytokine receptor interaction,” “phagosome” and “NF-kappa B signaling” (Fig. 3). Our findings are consistent with previous studies, and show that the pathways most significantly enriched in TB patients relative to healthy individuals were related to hematopoiesis and inflammation.^[33]^

Our findings were in agreement with the previous finding that DMTB patients show increased inflammatory responses^[2,33,35,36]^ compared to TB patients: expression levels of genes in multiple inflammatory pathways or activities, namely “myeloid leukocyte activation,” “signaling by interleukins,” “leukocyte differentiation” and “IL-17 signaling” increased in DMTB patients relative to TB patients (Fig. 4C). In addition, the expression of genes related to immunological activities such as “lymphocyte activation,” “PID CD8 TCR pathway,” lymphocyte differentiation’, and “natural killer cell mediated cytotoxicity” decreased in DMTB patients relative to TB patients, possibly explaining the increased susceptibility to TB and disease severity in the DMTB comorbidity (Fig. 4B). The hemostasis pathway was also enriched in groups of DEGs which displayed increased expression in TB-DM patients relative to TB patients (cluster 3, Fig. 4C) and in the 952 DEGs that uniquely present in DMTB patients relative to the controls (Fig. 5B). This pathway has been found to associate with granuloma necrosis leading to exacerbated lung damage in DMTB patients. Our results provide additional evidence that hemostasis is a risk factor for severe lung lesions in TB patients with DM.^[34]^

Our clustering and enrichment analysis yielded several additional interesting findings. Firstly, “Ras protein signal transduction” was enriched in cluster 2 and “small GTPase signal transduction” was enriched in the DMTB unique DEGs, suggesting that certain small GTPases may play a role in the pathogenesis of the DMTB comorbidity. It has been reported that defects in the signaling of small G-proteins such as Rac1 and Cdc42 can affect insulin secretion,^[37]^ and small GTPases such as Arl8b, Rac1 and several Ras-associated small GTPases have critical functions in the host immune response against tuberculosis.^[38–40]^ Therefore it is highly possible that small GTPases may be involved in the pathogenesis of DMTB. Our data identified several genes in this pathway, such as *RAC1, CDC42EP3, RASSF1* and *RASGEF1B*, that may be implicated in the DMTB comorbidity. Secondly, “protein kinase C signaling” was enriched in cluster 2, *PRKCZ, PLA2G6, DGKQ, FLT4* and *ADGRG1* being the enriched DEGs in this pathway. Upon *M. tuberculosis* infection, the rapid differential methylation of the enhancer region of *PRKCZ* (encoding protein kinase C (PKC) zeta) plays an important regulatory role in the transcriptional response to infection.^[41,42]^ *PLA2G6* encodes A2 phospholipase and activation of this enzyme is critically involved in the process of *M. tuberculosis* infection induced macrophage apoptosis.^[43]^ Specific pathological mechanisms of these molecules in the PKC signaling pathway in DMTB warrant further investigation. Last but not least, the cell cycle pathway was enriched in both cluster 3 and in the 952 DEGs uniquely found in DMTB profiles, implying that abnormal immune cell cycle progression may also underpin the DMTB comorbidity; the specific inflammatory or immunological components affected should be further investigated.

It should be noted that the sample size of our study was small, and confounding factors such as body mass index, and tobacco or alcohol consumption were not included or analyzed. Future studies with larger sample sizes and adjustments for more confounding factors will be needed to validate these findings.

Our study investigated the impact of diabetes on the pathogenesis of the DMTB comorbidity through transcriptome sequencing, providing useful information for studying the etiological mechanisms and developing new therapeutics against the menace of coincident diabetes and TB.

## Acknowledgements

We would like to gratefully acknowledge TB healthcare Co., Ltd. for the kind technical help.

## Author contributions

**Conceptualization:** Wenxu Hong, Tao Liu.

**Data curation:** Jing Gui.

**Formal analysis:** Yu Fu.

**Funding acquisition:** Wenxu Hong.

**Investigation:** Tao Liu, Yaguo Wang.

**Methodology:** Tao Liu, Yaguo Wang.

**Supervision:** Chunli Ye.

**Validation:** Xiangya Hong.

**Visualization:** Ling Chen, Yuhua Li.

**Writing—original draft:** Tao Liu, Yaguo Wang.

**Writing—review & editing:** Wenxu Hong.

## References

[1] WHO. Global tuberculosis report. 2018. Available at: https://apps.who.int/iris/bitstream/handle/10665/274453/9789241565646-eng.pdf.

[2] Venketaraman V. Understanding the Host Immune Response Against Mycobacterium Tuberculosis Infection. Springer. 2018.

[3] Dooley KE Chaisson RE. Tuberculosis and diabetes mellitus: convergence of two epidemics. Lancet Infect Dis. 2009;9:737–46. 19926034 10.1016/S1473-3099(09)70282-8 PMC2945809

[4] Restrepo BI. Diabetes and tuberculosis. Microbiol Spectr. 2016;4:10. 10.1128/microbiolspec.TNMI7-0023-2016 PMC5240796 28084206

[5] Zheng C Hu M Gao F. Diabetes and pulmonary tuberculosis: a global overview with special focus on the situation in Asian countries with high TB-DM burden. Glob Health Action. 2017;10:1–11. 10.1080/16549716.2016.1264702 PMC5328328 28245710

[6] Ugarte-Gil C Alisjahbana B Ronacher K. Diabetes mellitus among pulmonary tuberculosis patients from 4 tuberculosis-endemic countries: the TANDEM study. Clin Infect Dis. 2020;70:780–8. 30958536 10.1093/cid/ciz284

[7] Sireesha T Asha S Malathi J. Surreptitious TB infections with recently identified DM people: a cross- sectional study. Infect Disord Drug Targets. 2019;19:185–92. 30318005 10.2174/1871526518666181011152914

[8] Al-Rifai RH Pearson F Critchley JA. Association between diabetes mellitus and active tuberculosis: a systematic review and meta-analysis. PLoS One. 2017;12:e0187967. 29161276 10.1371/journal.pone.0187967 PMC5697825

[9] Sane Schepisi M Navarra A Altet Gomez MN. Burden and characteristics of the comorbidity tuberculosis-diabetes in Europe: TBnet prevalence survey and case-control study. Open Forum Infect Dis. 2019;6:ofy337. 30697572 10.1093/ofid/ofy337 PMC6330516

[10] Pande T Huddart S Xavier W. Prevalence of diabetes mellitus amongst hospitalized tuberculosis patients at an Indian tertiary care center: a descriptive analysis. PLoS One. 2018;13:e0200838. 30021016 10.1371/journal.pone.0200838 PMC6051633

[11] Chang JT Dou HY Yen CL. Effect of type 2 diabetes mellitus on the clinical severity and treatment outcome in patients with pulmonary tuberculosis: a potential role in the emergence of multidrug-resistance. J Formos Med Assoc. 2011;110:372–81. 21741005 10.1016/S0929-6646(11)60055-7

[12] Hodgson K Morris J Bridson T. Immunological mechanisms contributing to the double burden of diabetes and intracellular bacterial infections. Immunology. 2015;144:171–85. 25262977 10.1111/imm.12394 PMC4298412

[13] Jeon CY Murray MB Baker MA. Managing tuberculosis in patients with diabetes mellitus: why we care and what we know. Expert Rev Anti Infect Ther. 2012;10:863–8. 23030325 10.1586/eri.12.75

[14] Baker MA Lin HH Chang HY. The risk of tuberculosis disease among persons with diabetes mellitus: a prospective cohort study. Clin Infect Dis. 2012;54:818–25. 22238171 10.1093/cid/cir939

[15] Jimenez-Corona ME Cruz-Hervert LP Garcia-Garcia L. Association of diabetes and tuberculosis: impact on treatment and post-treatment outcomes. Thorax. 2013;68:214–20. 23250998 10.1136/thoraxjnl-2012-201756 PMC3585483

[16] Critchley JA Restrepo BI Ronacher K. Defining a research agenda to address the converging epidemics of tuberculosis and diabetes: Part 1: epidemiology and clinical management. Chest. 2017;152:165–73. 28434936 10.1016/j.chest.2017.04.155 PMC5989639

[17] Ahmad SR Yaacob NA Jaeb MZ. Effect of diabetes mellitus on tuberculosis treatment outcomes among tuberculosis patients in Kelantan, Malaysia. Iran J Public Health. 2020;49:1485–93. 33083325 10.18502/ijph.v49i8.3892 PMC7554400

[18] Baker MA Harries AD Jeon CY. The impact of diabetes on tuberculosis treatment outcomes: a systematic review. BMC Med. 2011;9:81. 21722362 10.1186/1741-7015-9-81 PMC3155828

[19] Restrepo BI Twahirwa M Rahbar MH. Phagocytosis via complement or Fc-gamma receptors is compromised in monocytes from type 2 diabetes patients with chronic hyperglycemia. PLoS One. 2014;9:e92977. 24671137 10.1371/journal.pone.0092977 PMC3966862

[20] Giacco F Brownlee M. Oxidative stress and diabetic complications. Circ Res. 2010;107:1058–70. 21030723 10.1161/CIRCRESAHA.110.223545 PMC2996922

[21] Lagman M Ly J Saing T. Investigating the causes for decreased levels of glutathione in individuals with type II diabetes. PLoS One. 2015;10:e0118436. 25790445 10.1371/journal.pone.0118436 PMC4366217

[22] Kim D Langmead B Salzberg SL. HISAT: a fast spliced aligner with low memory requirements. Nat Methods. 2015;12:357–60. 25751142 10.1038/nmeth.3317 PMC4655817

[23] Trapnell C Williams BA Pertea G. Transcript assembly and quantification by RNA-Seq reveals unannotated transcripts and isoform switching during cell differentiation. Nat Biotechnol. 2010;28:511–5. 20436464 10.1038/nbt.1621 PMC3146043

[24] Roberts A Pimentel H Trapnell C. Identification of novel transcripts in annotated genomes using RNA-Seq. Bioinformatics. 2011;27:2325–9. 21697122 10.1093/bioinformatics/btr355

[25] Mortazavi A Williams BA McCue K. Mapping and quantifying mammalian transcriptomes by RNA-Seq. Nat Methods. 2008;5:621–8. 18516045 10.1038/nmeth.1226 PMC13303166

[26] Anders S Huber W. Differential Expression of RNA-Seq Data at the Gene Level – The DESeq Package. Heidelberg, Germany: European Molecular Biology Laboratory (EMBL). 2012;10:f1000research.

[27] Kolde R. Pheatmap: Pretty Heatmaps, R Package Version 1.0.12. 2015. Available at: https://cran.r-project.org/web/packages/pheatmap/index.html [access date March 20, 2021].

[28] Tusher VG Tibshirani R Chu G. Significance analysis of microarrays applied to the ionizing radiation response. Proc Natl Acad Sci USA. 2001;98:5116–21. 11309499 10.1073/pnas.091062498 PMC33173

[29] Zhou Y Zhou B Pache L. Metascape provides a biologist-oriented resource for the analysis of systems-level datasets. Nat Commun. 2019;10:1523. 30944313 10.1038/s41467-019-09234-6 PMC6447622

[30] Doncheva NT Morris JH Gorodkin J. Cytoscape StringApp: network analysis and visualization of proteomics data. J Proteome Res. 2019;18:623–32. 30450911 10.1021/acs.jproteome.8b00702 PMC6800166

[31] Li N Li L Chen Y. The Identification of core gene expression signature in hepatocellular carcinoma. Oxid Med Cell Longev. 2018;2018:3478305. 29977454 10.1155/2018/3478305 PMC5994271

[32] Kumar L Futschik ME. Mfuzz: a software package for soft clustering of microarray data. Bioinformation. 2007;2:5–7. 18084642 10.6026/97320630002005 PMC2139991

[33] Joosten SA Fletcher HA Ottenhoff TH. A helicopter perspective on TB biomarkers: pathway and process based analysis of gene expression data provides new insight into TB pathogenesis. PLoS One. 2013;8:e73230. 24066041 10.1371/journal.pone.0073230 PMC3774688

[34] Dong Z Shi J Dorhoi A. Hemostasis and lipoprotein indices signify exacerbated lung injury in TB with diabetes comorbidity. Chest. 2018;153:1187–200. 29224833 10.1016/j.chest.2017.11.029

[35] Ronacher K Joosten SA van Crevel R. Acquired immunodeficiencies and tuberculosis: focus on HIV/AIDS and diabetes mellitus. Immunol Rev. 2015;264:121–37. 25703556 10.1111/imr.12257

[36] Kumar NP Banurekha VV Nair D. Coincident pre-diabetes is associated with dysregulated cytokine responses in pulmonary tuberculosis. PLoS One. 2014;9:e112108. 25393696 10.1371/journal.pone.0112108 PMC4230980

[37] Kowluru A. Role of G-proteins in islet function in health and diabetes. Diabetes Obes Metab. 2017;19(Suppl 1):63–75. 28880478 10.1111/dom.13011 PMC5657296

[38] Michelet X Tuli A Gan H. Lysosome-mediated plasma membrane repair is dependent on the small GTPase Arl8b and determines cell death type in mycobacterium tuberculosis infection. J Immunol. 2018;200:3160–9. 29592961 10.4049/jimmunol.1700829 PMC5995332

[39] Jacobsen M Repsilber D Gutschmidt A. Ras-associated small GTPase 33A, a novel T cell factor, is down-regulated in patients with tuberculosis. J Infect Dis. 2005;192:1211–8. 16136464 10.1086/444428

[40] Sun J Singh V Lau A. Mycobacterium tuberculosis nucleoside diphosphate kinase inactivates small GTPases leading to evasion of innate immunity. PLoS Pathog. 2013;9:e1003499. 23874203 10.1371/journal.ppat.1003499 PMC3715411

[41] Pacis A Tailleux L Morin AM. Bacterial infection remodels the DNA methylation landscape of human dendritic cells. Genome Res. 2015;25:1801–11. 26392366 10.1101/gr.192005.115 PMC4665002

[42] Cizmeci D Dempster EL Champion OL. Mapping epigenetic changes to the host cell genome induced by Burkholderia pseudomallei reveals pathogen-specific and pathogen-generic signatures of infection. Sci Rep. 2016;6:30861. 27484700 10.1038/srep30861 PMC4971488

[43] Duan L Gan H Arm J. Cytosolic phospholipase A2 participates with TNF-alpha in the induction of apoptosis of human macrophages infected with Mycobacterium tuberculosis H37Ra. J Immunol. 2001;166:7469–76. 11390500 10.4049/jimmunol.166.12.7469

